# Biomolecular Characterization of Putative Antidiabetic Herbal Extracts

**DOI:** 10.1371/journal.pone.0148109

**Published:** 2016-01-28

**Authors:** Verena Stadlbauer, Renate Haselgrübler, Peter Lanzerstorfer, Birgit Plochberger, Daniela Borgmann, Jaroslaw Jacak, Stephan M. Winkler, Klaus Schröder, Otmar Höglinger, Julian Weghuber

**Affiliations:** 1 University of Applied Sciences Upper Austria, Wels, Austria; 2 University of Applied Sciences Upper Austria, Linz, Austria; 3 University of Applied Sciences Upper Austria, Hagenberg, Austria; 4 BioMed zet Life Science GmbH, Linz, Austria; 5 Vienna University of Technology, Vienna, Austria; GDC, GERMANY

## Abstract

Induction of GLUT4 translocation in the absence of insulin is considered a key concept to decrease elevated blood glucose levels in diabetics. Due to the lack of pharmaceuticals that specifically increase the uptake of glucose from the blood circuit, application of natural compounds might be an alternative strategy. However, the effects and mechanisms of action remain unknown for many of those substances. For this study we investigated extracts prepared from seven different plants, which have been reported to exhibit anti-diabetic effects, for their GLUT4 translocation inducing properties. Quantitation of GLUT4 translocation was determined by total internal reflection fluorescence (TIRF) microscopy in insulin sensitive CHO-K1 cells and adipocytes. Two extracts prepared from purslane (*Portulaca oleracea*) and tindora (*Coccinia grandis*) were found to induce GLUT4 translocation, accompanied by an increase of intracellular glucose concentrations. Our results indicate that the PI3K pathway is mainly responsible for the respective translocation process. Atomic force microscopy was used to prove complete plasma membrane insertion. Furthermore, this approach suggested a compound mediated distribution of GLUT4 molecules in the plasma membrane similar to insulin stimulated conditions. Utilizing a fluorescent actin marker, TIRF measurements indicated an impact of purslane and tindora on actin remodeling as observed in insulin treated cells. Finally, *in-ovo* experiments suggested a significant reduction of blood glucose levels under tindora and purslane treated conditions in a living organism. In conclusion, this study confirms the anti-diabetic properties of tindora and purslane, which stimulate GLUT4 translocation in an insulin-like manner.

## Introduction

Globally, as of 2013, an estimated 381 million people had diabetes mellitus. Its prevalence has dramatically increased and results in a considerably higher rate of mortality worldwide [1]. Type 2 diabetes mellitus (T2DM) makes up about 90% of all cases and is caused by a combination of inadequate compensatory insulin secretion and a lack of functional insulin receptors, termed insulin resistance [2]. Most importantly, insulin induces the uptake of glucose by adipocytes and muscle cells via insulin receptor signaling: Activation and auto-phosphorylation of the receptor leads to recruitment of various intracellular binding partners including insulin receptor substrates [3], activation of protein kinase B (Akt) via PIP_3_ formation [4], and finally the translocation of glucose transporter 4 (GLUT4) from intracellular storage compartments to the plasma membrane [5]. Insulin-dependent GLUT4 translocation is insufficient when insulin resistance occurs, leading to reduced glucose uptake and the elevation of blood glucose levels. Commonly used therapeutic drugs for the treatment of T2DM produce side effects including fat accumulation or defective hepatic regeneration [6, 7]. Therefore, there is a need to design novel insulin-mimetic products that stimulate GLUT4 translocation and subsequent glucose uptake without side effects. Natural compounds have been discussed as a valuable alternative to synthetic therapeutic drugs in this regard. Products that have been considered having great potential include especially plants with a high concentration of polyphenolic compounds [8–12], and other characteristic substances such as alkaloids [13], or alpha lipoic acid [14].

A promising plant with reported anti-diabetic properties is purslane (*Portulaca oleracea*), which has been used as folk medicine for the treatment of diabetes mellitus for many years [15]. The molecular mode of action remains unclear, however, studies indicated a blood glucose, plasma triglyceride, and plasma LDL-cholesterol reduction, reduced vascular inflammation, as well as an increased insulin secretion [16–18]. Polyunsaturated fatty acids, flavonoids and polysaccharides found in purslane have been linked to the aforementioned effects [15]. Bilberry (*Vaccinium myrtillus*) has been described as another anti-diabetic plant containing high levels of polyphenolic compounds. While some studies could not prove an anti-hyperglycemic effect [19], other groups reported on a positive effect on glucose homeostasis in diabetic mice [20]. Clinical studies performed with ginger (*Zingiber officinale*) and tindora (*Coccinia grandis*) indicated decreased blood glucose levels in humans and animals [21–24]. Finally, there is an evidence that natural products of the putative anti-diabetic plants milk-thistle (*Silybum marianum*), and jiaogulan (*Gynostemma pentaphyllum*) stimulate insulin secretion, inhibit gluconeogenesis, or reduce triglycerides and insulin resistance [25–27].

Importantly, knowledge on the effectiveness of natural compounds on a molecular level is still very limited and mainly supported by wet-lab chemistry based approaches performed with several different types of cell lines. The aim of this work was to better understand the molecular mode of action of selected putative insulin mimetic substances using established cell models in combination with fluorescence microscopy, atomic force microscopy, and glucose uptake assays. We especially focused on the GLUT4 translocation, plasma membrane insertion, and actin remodeling promoting activity of the tested substances in living cells, and if this effect really correlates with elevated intracellular glucose concentrations. In addition, we utilized a chicken embryo model (HET-CAM) to prove if an increased cellular glucose uptake mediated by natural insulin mimetic compounds *in-vitro* also leads to reduced blood glucose levels in a living organism. Our results confirm the potential of some substances in this regard and gain insight into the mode of action on a molecular level.

## Experimental Procedures

### DNA constructs and chemicals

The pLifeact-tdTomato construct was kindly provided by Weiping Han (Laboratory of Metabolic Medicine, Singapore). Human insulin, insulin from bovine pancreas, dexamethasone, 3-isobutyl-1-methylxanthine, wortmannin, SB203518, dorsomorphin (compound C), polyphenon 60 from green tea (PP60), HEPES, CaCl_2_, NaCl, KCl, MgSO_4_ and KH_2_PO_4_ were purchased from Sigma-Aldrich (Schnelldorf, Germany). Mouse anti-myc monoclonal antibody (9E10: sc-40 and sc-40 AF647) was from Santa Cruz biotechnology (Santa Cruz, CA). Bilberry (BIL, *Vaccinium myrtillus*) extract was provided by Vitalingo (Berlin, Germany), ginger (GIN, *Zingiber officinale*) extract by Hawlik Gesundheitsprodukte (Strasslach, Germany), Tindora (TIN, *Coccinia grandis*) extract by SHAG psoriasisEX (Berlin, Germany), milk thistle extract (MT, *Silybum marianum*) by McVital (Newark, USA), purslane (PUR, *Portulaca oleracea*) extract by Frutarom (Portusana^®^ EFLA308, Haifa, Israel), and jiaogulan (JIA, *Gynostemma pentaphyllum*) extract by Vitabay (Kerkrade, Netherlands). For preparation of stock solutions, the herbal compounds were dissolved in Krebs Ringer Phosphate HEPES buffer (KRPH; 20 mM HEPES, 1 mM CaCl_2_, 136 mM NaCl, 4.7 mM KCl, 1 mM MgSO_4_ and 5 mM KH_2_PO_4_) (PUR and PP60) or in 50% DMSO and 50% KRPH buffer (TIN, GIN, BIL, JIA, MT).

### Cell Culture and Transfection

CHO-K1 cells stably expressing hIR and GLUT4-myc-GFP were a kind gift from Manoj K. Bhat (National Centre for Cell Science, University of Pune, India).[28] CHO-K1 hIR/GLUT4-myc-GFP cells were maintained in Ham’s F12 culture medium supplemented with 100 μg/ml penicillin, 100 μg/ml streptomycin, 1% G418 and 10% fetal bovine serum (FBS) (all Life Technologies, Carlsbad, CA), and grown in a humidified atmosphere at 37°C and 5% CO_2_. Transient expression of pLifeact-tdTomato in CHO-K1 hIR/GLUT4-myc-GFP cells was achieved by electroporation using an Amaxa/Lonza nucleofector device (Lonza, Basel, Switzerland) according to the manufacturer (Kit T). In short, 1x10^6^ CHO-K1 hIR/GLUT4-myc-GFP cells were transfected at 50–70% confluence with 0.18 μg DNA. Transfected cells were grown in 96 well imaging plates (Nunc, Roskilde, Denmark) for 24 hours prior imaging. 3T3-L1 cells were purchased from ATCC (Manassas, USA). 3T3-L1 cells stably expressing GLUT4-GFP were obtained from Alan Saltiel (University of Michigan). Both cell lines were maintained in DMEM high glucose culture medium supplemented with 100 μg/ml penicillin, 100 μg/ml streptomycin and 10% newborn calf serum (NCS) (all Life Technologies, Carlsbad, USA), and grown in a humidified atmosphere at 37°C and 5% CO_2_.

### TIRF microscopy

CHO-K1 cells were grown in 96-well imaging plates (35,000 cells/well; Nunc, Roskilde, Denmark) over night. Cell culture medium was aspirated off and, after washing the cells with PBS (VWR, Vienna, Austria), replaced by HBSS (Thermo Fisher, Waltham, MA) supplemented with 0.1% BSA (Sigma-Aldrich, Schnelldorf, Germany) for 3 hours. 3T3-L1 cells were seeded in 96-well imaging plates (20,000 cells/well) and cultured for 24 hours. The culture medium was aspirated off and, after washing the cells with PBS, replaced by serum-free culture medium overnight. Cells were incubated with insulin or herbal compounds dissolved in KRPH buffer and imaged on an Olympus IX-81 inverted microscope in objective-type TIR configuration via an Olympus 60x NA = 1.49 Plan-Apochromat objective. 96-well plates were placed on an x-y-stage (CMR-STG-MHIX2-motorized table; Märzhäuser, Wetzlar, Germany). Scanning of larger areas was supported by a laser-guided automated focus-hold system (ZDC2). The 488 nm, 561 nm and 647 nm emission of diode lasers (Toptica Photonics, Munich, Germany) were used to image GFP, tdTomato and Alexa647 fluorescence, respectively. After appropriate filtering, the fluorescence signal was recorded via an Orca-R2 CCD camera (Hamamatsu Photonics, Herrsching, Germany).

### Immunofluorescence

Cells grown in 96-well imaging plates were starved in HBSS buffer for 3 hours and incubated with insulin or herbal compounds for 10 minutes. Afterwards the cells were fixed with 4% precooled para-formaldehyde (Sigma-Aldrich, Schnelldorf, Germany) for 15 minutes on ice followed by two washing steps with PBS and a blocking step with 5% FBS and 5% BSA in PBS for one hour at room temperature. Blocking solution was replaced by 4 μg/ml anti-myc Alexa647 antibody diluted in PBS for one hour. Prior to the microscopy experiments three additional PBS washing steps were carried out.

### Correlated epi-fluorescence/force spectroscopy measurements

CHO-K1 hIR/GLUT4-myc-GFP cells were grown on 30 mm coverslip glass-slides (350,000 cells/slide) over-night, starved for 3 hours in HBSS buffer, stimulated with the indicated substance for 10 minutes, fixed with 4% precooled para-formaldehyde for 15 minutes, and washed twice with PBS buffer. AFM measurements were performed using a NanoWizard 3 (JPK Instruments AG, Berlin, Germany) system mounted on an Axiovert 200 inverted microscope (Carl Zeiss AG, Oberkochen, Germany). The microscope is equipped with a 100x NA = 1.46 oil-immersion Plan-Apochromat TIRFM objective (Zeiss, Oberkochen, Germany) and a 20x NA = 0.8 Plan-Apochromat objective (Carl Zeiss AG, Oberkochen, Germany). Samples were illuminated in epifluorescence configuration via the epiport using 488 nm (250 mW) light from a diode laser (Toptica Photonics, Munich, Germany), which allows for precise control of the illumination timings via direct modulation of TTL pulses. After appropriate filtering, emitted signals were imaged on a back-illuminated, TE-cooled CCD-camera (Andor iXon Ultra 897, Belfast, UK). Timing protocols were generated by an in-house program package implemented in LABVIEW (National Instruments, Austin, TX, USA). Illumination times were adjusted to values between 1 and 5 ms. The sample surface was first imaged with the fluorescence microscope to determine an appropriate site for the force curve measurements. Topographical images and force spectroscopy data were recorded either in QI^™^ mode (Quantitative Imaging mode) or in Force Mapping mode at room temperature in liquid (PBS) at a resolution of 32 x 32 pixels. The maximum force determined by the vertical deflection of the cantilever was set to 250 pN. Force distance cycles (scan rates) were controlled by the z length (1,000 nm) with a speed of 2 μm/s in closed loop configuration. We used uncoated silicon cantilevers (MSNL-10, Bruker Corporation, Billerica, USA) with a nominal spring constant in the range of 0.01–0.02 N/m. JPK data processing (JPK Instrument, Berlin, Germany) software was used for visualization of topographical images and for force curve processing. The height, adhesion and slope of the force curve were collected simultaneously in both trace and retrace directions. The unbinding force was estimated from force curve by using the worm-like chain (WLC) model provided by JPK data processing software.

### 2-DG uptake assay

Glucose uptake experiments were conducted according to the manufacturer’s instructions (Glucose Uptake Colorimetric Assay Kit, Sigma Aldrich, Schnelldorf, Germany). Briefly, 3T3-L1 cells were differentiated after seeding 2,000 cells per well into 96 well tissue culture plates (Greiner, Kremsmünster, Austria). Confluent cells were further cultivated for 48 hours, and growth medium was replaced by differentiation medium (DMEM high glucose culture medium supplemented with 100 μg/ml penicillin, 100 μg/ml streptomycin, 10% FBS, 1 μM dexamethasone, 0.5 mM IBMX and 1 μg/ml insulin from bovine pancreas) for further 48 hours. Finally, cells were cultured in adipocyte maintenance medium (DMEM high glucose culture medium supplemented with 100 μg/ml penicillin, 100 μg/ml streptomycin, 10% FBS, and 1 μg/ml insulin) for 7–10 days. Medium was changed every 48 hours. Finally, medium was replaced by serum-free medium overnight followed by 40 minutes incubation in KRPH-buffer. Cells were then stimulated with indicated compounds (100 μl/well) for 20 minutes and 10 μl of 10 mM 2-DG was added for further 20 minutes. Cells were washed 3 times with PBS, lysed with 80 μl of extraction buffer, frozen at -80°C for 5 minutes and heated at 85°C for 40 minutes. The lysates were cooled down on ice for 5 minutes and neutralized by adding 10 μl of neutralization buffer. Insoluble material was removed by spinning down the extract at 15,000 rpm for 5 minutes and the lysate was then diluted 1:10 in assay buffer. Finally, TNB generation was set up by two amplification steps according to instructions. Absorbance was measured at 412 nm on a plate reader (POLARstar omega, BMG LABTECH, Ortenberg, Germany). Each sample was measured at least 3 times in duplicates.

### Hens egg chorioallantoic membrane test (HET-CAM)

Fertilized eggs (Lohmann classic brown chicken) were incubated for 10 days at 37°C with an average humidity around 40%. The eggs were constantly turned automatically. Eggs were checked for fertilization via candling and the area of the air bladder was marked. The eggshell was lightly pecked with a pointed pair of tweezers in this area and 300 μl of the substance were added with a syringe. NovoRapid and purslane extract were dissolved in KRPH buffer. Tindora was dissolved in KRPH buffer supplemented with 2% DMSO. After incubation for different time intervals (30 min up to 3 hours) at 37°C, the eggshell above the air bladder was carefully removed and the eggshell membrane was equilibrated with PBS. In a next step the eggshell membrane was removed and the chorioallantoic membrane was carefully cut with a micro-scissor. A suitable blood vessel was placed and cut on a pH strip, the blood was collected with a pipette, coagulated at room temperature and centrifuged for 15 min at 15,000 rpm. Plasma glucose levels were determined by HPLC analysis. Therefore, a Jasco RI-2031 Plus detector and a UV-Vis detector equipped with Chrompass software (Jasco Corporation, Tokyo, Japan) were used. The column (Macherey-Nagel, Düren, Germany) temperature was 65°C, and 5 mM H_2_SO_4_ was used as an eluent with an average flow of 0.8 ml/min. For each time point at least 10 fertilized eggs were used. Each experiment was repeated at least three times. The applicability of the investigated substances was confirmed by toxicity tests. Therefore, 300 μl of the respective substance at different concentrations (100–10,000 mg/l) were added into the air bladder at day 9. At this development stage the nervous system is not completed, and it is thus secured that the embryo does not suffer from pain. The eggs were further incubated at 37°C for 24 hours, opened, and the embryos were checked for vitality. The rapid loss of blood during blood collection (about 400 μl per fertilized egg) led to an immediate death of the chicken embryo. No additional manipulations to the fertilized eggs occurred after the 10 day incubation time. The HET-CAM test, which has been validated by an EU Directive on dangerous substances, is an alternative to tests on live animals such as the Draize rabbit eye test. The approval by an ethics committee is not required, since the HET-CAM test is not an animal experiment in a legal sense.

### Data analysis

Initial imaging recordings were supported by the Olympus Xcellence RT software. In-depth analysis for the calculation of the fluorescence intensity in individual cells and a fast comparison of the fluorescent signal in numerous cells at different time intervals was performed using the *Spotty* framework. Spotty can be retrieved online at http://bioinformatics.fh-hagenberg.at/projects/microprot/. The most important analysis algorithms integrated in the intensity analysis in *Spotty* are: a) preprocessing (including correlation based optimal downsampling, filtering and creation of layer-based images), b) cell detection and c) results analysis. Statistical analysis was carried out using unpaired t test in Graphpad Prism (version 6.02).

## Results

### Induction of GLUT4 translocation by herbal compounds

**Fig 1:**
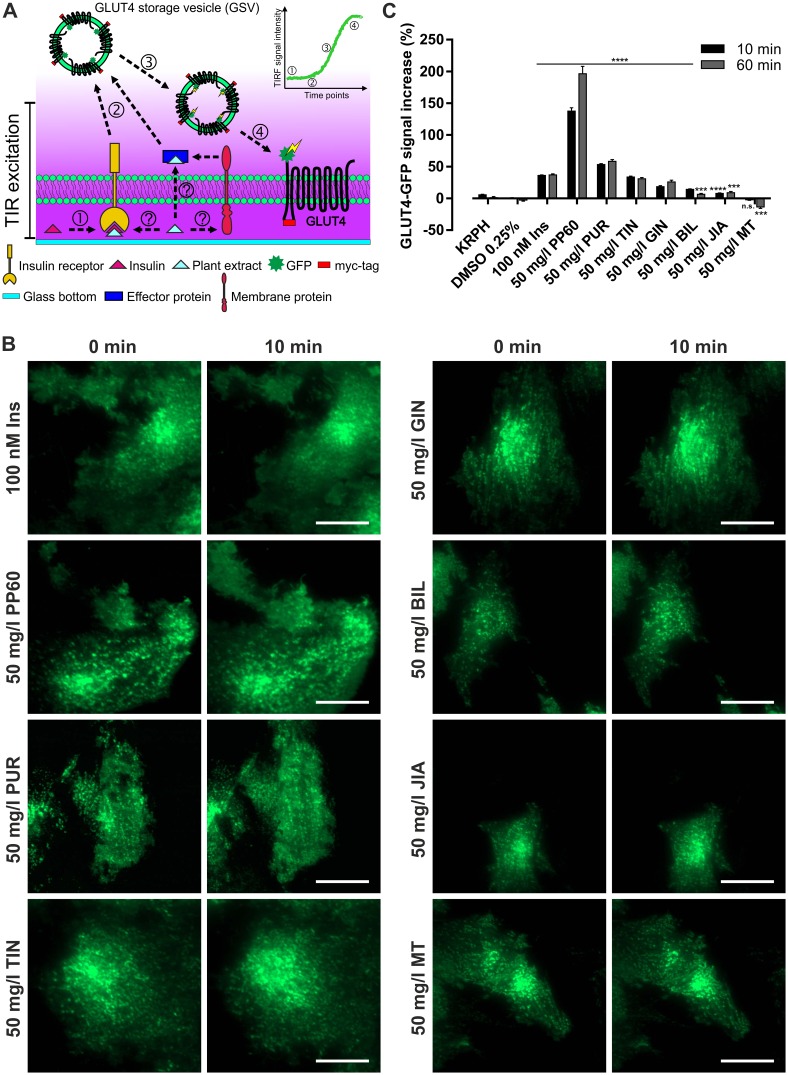
GLUT4 translocation induction by putative insulin mimetic herbal compounds quantitated by TIRF microscopy. (A) Schematic illustration of insulin or herbal compound induced GLUT4 translocation. Putative insulin mimetic substances stimulating the insulin receptor (IR), other plasma membrane or cytosolic effector proteins (1) induce signal transduction events (2) that lead to the translocation of GLUT4 containing vesicles (3) and their subsequent fusion with the PM (4). This transfer is linked to an increase of the GFP-signal intensity in the evanescent field. (B) GLUT4-GFP signal in CHO-K1 hIR/GLUT4-myc-GFP cells before and after stimulation with indicated substances. Cells were seeded in 96-well plates (35,000 cells/well) and grown over night followed by 3 hours of starvation in HBSS buffer. Cells were then stimulated by the indicated substances for 10 min and the GFP-signal was recorded. Scale bar = 20 μm. (C) The GLUT4-GFP signal intensity increase in the evanescent field was quantitated after 10 and 60 minutes. Error bars are based on the standard error of the mean. At least 60 cells were analyzed for each substance. Data were collected from the same cells before and after substance application. ***P < 0.001 and ****P < 0.0001, significant increase with respect to KRPH (Ins, PP60, PUR) or 0.25% DMSO in KRPH (TIN, GIN, BIL, JIA, MT) treated cells, respectively.

We have recently shown that TIRF microscopy is a superior tool for the quantitation of GLUT4 translocation in living cells [29]. Here, we used this approach to determine the effects of various herbal compounds on this translocation process. Therefore, CHO-K1 cells stably expressing the human insulin receptor and a GLUT4-myc-GFP fusion protein were starved and incubated with the chosen compound [29]. Subsequently, the increase of the fluorescence intensity in the evanescent field, which by trend correlates with an elevated GLUT4 translocation, was determined (Fig 1A).

Seven different plant extracts or compound mixtures that have been shown to influence GLUT4 translocation, intracellular glucose concentrations, or blood glucose levels were chosen for this study. In a first attempt we exclusively focused on the GLUT4 GFP-signal (Fig 1B). In comparison to the treatment with 100 nM insulin, which led to an increase of the GFP-signal of ~36% within 10 minutes, incubation with 50 mg/l purslane extract or the polyphenol mixture (PP60) for the same time period resulted in a major increase (~53% and ~137%, respectively; Fig 1C). Treatment of the cells with 50 mg/l tindora or ginger extract led to a moderate increase (~34% and ~18%, respectively), while 50 mg/l bilberry, jiaogulan, or milk thistle extract resulted in an increase near or below the chosen threshold value of 10%. Similar to insulin, elevated GFP-signals were found to be constant for 60 minutes for the herbal compounds.

### Extent of GLUT4 plasma membrane insertion upon treatment with herbal compounds

**Fig 2:**
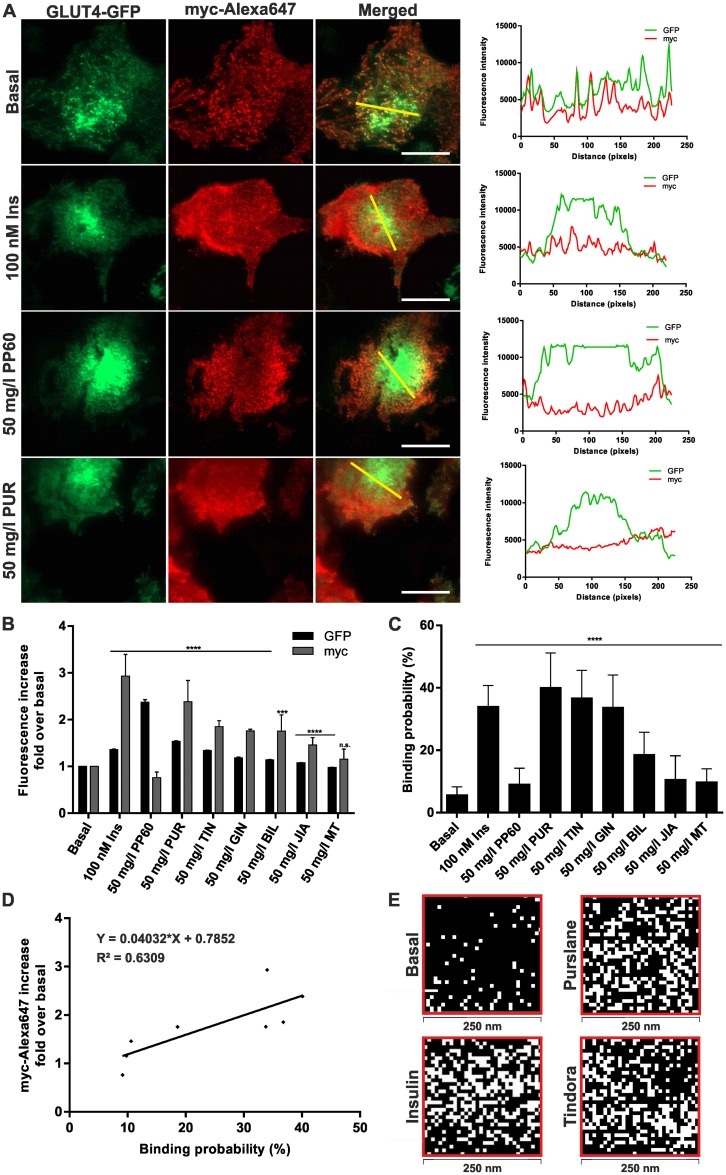
Quantitation of GLUT4 plasma membrane insertion. (A) Representative TIRF microscopy images and fluorescence intensity profiles for the indicated line scans (marked in yellow in the merged images) of GLUT4-GFP and Alexa647-anti-myc staining under basal and compound-treated conditions. CHO-K1 hIR/GLUT4-myc-GFP cells were grown over night in 96-well plates (35,000 cells/well), starved for 3 hours in HBSS buffer, stimulated for 10 minutes, fixed with 4% para-formaldehyde, and stained using an Alexa647 labeled anti-myc antibody. Fluorescence in the evanescent field was recorded for GFP and Alexa647 at 488 and 640 nm, respectively. Scale bar = 20 μm. (B) Quantitation of fluorescence signals in stimulated cells with respect to untreated cells (n > 120 cells). Fluorescence was normalized to the values prior stimulation. Error bars are based on the standard error of the mean. ***P < 0.001 and ****P < 0.0001, significant increase with respect to starved cells. (C) Binding probability determined by fluorescence-guided AFM measurements under starved and compound treated conditions. Cells were grown on standard 30 mm glass slides (350,000 cells/slide) over night, starved for 3 hours in HBSS buffer, stimulated with the indicated substances for 10 minutes, and fixed with 4% para-formaldehyde. Error bars are based on the standard error of the mean. ****P < 0.0001, significant increase with respect to starved cells. (D) Linear correlation regression analysis between myc-staining and AFM measurements. (E) AFM mapping experiments of starved, insulin, purslane, ginger, and tindora treated cells, respectively. Force curves were acquired on a surface area of 250 nm x 250 nm with a step size of approximately 7–8 nm. White pixels represent positive unbinding events, whereas black pixels depict no binding.

An increase of the GFP-signal in the evanescent field is an important hint, but not an absolute proof for correct plasma membrane insertion of GLUT4. To further address this question we used the myc-tag in the first exofacial loop of the GLUT4 protein to prove that the detected increase of the GFP-signal is really due to an elevated insertion of the transporter into the plasma membrane [30]. Therefore, we starved CHO-K1 hIR/GLUT4-myc-GFP cells, induced GLUT4 translocation by insulin or the herbal compounds, fixed the cells, and labeled them with an anti-myc Alexa647 antibody. TIRF-microscopy was used to quantitate the fluorescence intensity of n > 100 cells for each sample in both color channels (Fig 2A). As shown in Fig 2B, we detected an increase of ~3-fold in the myc-channel upon insulin stimulation (100 nM for 10 minutes). Incubation with purslane extract resulted in the strongest increase of all tested herbal compounds (~2.4-fold), the one of tindora (~1.8-fold), ginger (~1.7-fold), and bilberry extract (~1.7-fold) was significantly lower. Addition of milk thistle and jiaogulan extract led to no or only a minor increase in the myc-channel. Interestingly, incubation with PP60, which was linked to the highest GFP-signal increase, did not show an increase in the myc-channel at all. To exclude autofluorescence of PP60, we incubated CHO-K1 cells stably expressing an insulin-insensitive plasma membrane protein (GPI-anchored GFP) [31] with PP60 and determined a potential increase of the GFP-signal in the evanescent field. As shown in S1A and S1B Fig no change was observed. This result indicates that plasma membrane insertion as the final step of GLUT4 translocation does not occur upon PP60 treatment.

To further confirm these findings we used fluorescence-guided AFM force measurements to detect GLUT4-myc-GFP molecules on the surface of CHO-K1 hIR/GLUT4-myc-GFP cells. For these experiments an anti-myc antibody was bound to the AFM tip to detect fully membrane-inserted GLUT4-myc-GFP molecules. This approach has recently been successfully applied to determine the reduced binding probability of starved cells in comparison to insulin treated cells [29]. As indicated in Fig 2C, we found an increased binding probability of cells treated with purslane extract (40%), tindora (37%), and ginger extract (34%). Incubation with PP60, bilberry, jiaogulan, and milk thistle extract led to binding probabilities comparable to or slightly higher than starved cells, which serves as a blocking control. Taken together, immunofluorescence experiments and force measurements gave similar results as confirmed by linear regression and correlation analysis (Fig 2D). In addition to the determination of binding probabilities, AFM offers the possibility to analyze the lateral distribution and clustering of proteins in the plasma membrane [32]. As shown in Fig 2E, mapping experiments indicate an increase of recorded unbinding events in the selected cell regions upon insulin treatment, and the detected binding signals appear randomly distributed. The same approach was used to study the distribution of GLUT4 proteins in the plasma membrane of cells treated with purslane, tindora, or ginger extract. The distribution of detected unbinding events appeared similar to the one of insulin stimulated cells. To further prove the random distribution of GLUT4 we used the Kolmogorov-Smirnov (KS) test, which is a suitable tool for the comparison of cluster size and distribution in microscopy images [33]. Cluster analysis showed that treatment of the cells with purslane or tindora extract has a similar effect on the distribution of GLUT4 molecules in the plasma membrane as insulin stimulation (data not shown).

### Induction of GLUT4 translocation and effects on intracellular glucose levels by herbal compounds in adipocytes

**Fig 3:**
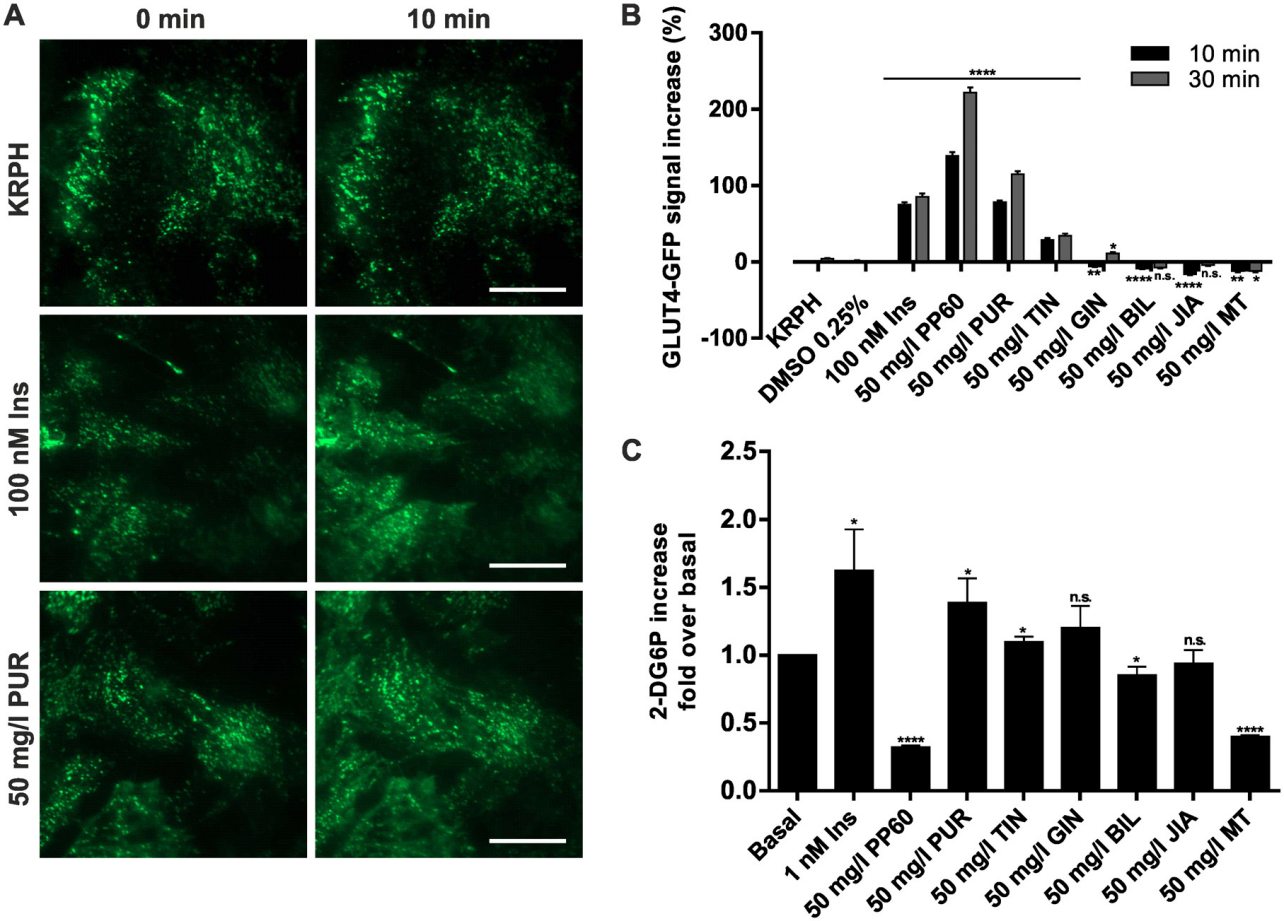
Quantitation of GLUT4 translocation and glucose uptake in 3T3-L1 cells. (A) TIRF microscopy based quantitation of GLUT4 translocation in 3T3-L1 GLUT4-GFP adipocytes. Cells were grown in 96-well plates (20,000 cells/well) for 24 hours and then starved overnight in serum-free medium. Images before and 10 minutes after stimulation with indicated substances are shown. Scale bar = 20 μm. (B) GLUT4-GFP signal intensity increase was analyzed after 10 and 30 minutes of stimulation. Error bars are based on the standard error of the mean. **P < 0.01, ***P < 0.001 and ****P < 0.0001, significant increase with respect to KRPH treated cells. (C) For glucose uptake measurements 3T3-L1 cells were grown in 96-well plates (2,000 cells/well) and differentiated to adipocytes. Cells were starved in serum-free medium overnight, glucose deprived in KRPH buffer for 40 minutes, and then stimulated with the indicated substances for 20 minutes followed by addition of 2-DG (20 minutes). Cell extracts were prepared and 2-DG uptake was measured after a colorimetric reaction using a plate reader device. Samples were measured in duplicates at least in three individual experiments. Error bars are based on the standard error of the mean. *P < 0.05, **P < 0.01 and ****P < 0.0001, significant increase with respect to KRPH (Ins, PP60, PUR) or 0.25% DMSO in KRPH (TIN, GIN, BIL, JIA, MT) treated cells.

CHO-K1 cells are not bona fide adipocytes and the signal transduction machinery essential for GLUT4 translocation might be incomplete. Therefore, to confirm the GLUT4 translocation inducing effects of selected plant extracts observed in CHO-K1 cells, we used TIRF microscopy to quantitate the translocation process in 3T3-L1 cells stably expressing GLUT4-GFP (Fig 3A). As shown in Fig 3B we detected a significant increase of the GFP-signal upon incubation with PP60, purslane, and tindora extract. A minor response for ginger extract was only found in cells incubated with this substance for 30 min. All other tested compounds as well as mock controls did not result in increased GFP-signals. In contrast, bilberry, jiaogulan, and milk thistle extract slightly reduced the fluorescence intensity in the GFP channel.

As a next step we quantitated the effects of the herbal compounds on the concentration of intracellular glucose levels in this cell line. Therefore, we used a 2-DG based glucose uptake assay kit to quantitate the glucose concentration of differentiated, starved and compound-treated cells. As indicated in Fig 3C we found a significant 2-DG6P increase of cells incubated with purslane extract and insulin (1.4-fold and 1.6-fold, respectively). Tindora and ginger extract led to a minor increase, while treatment with bilberry or jiaogulan extract resulted in a minor decrease, respectively. Incubation with milk thistle extract or PP60 was found to significantly decrease the 2-DG6P concentration (1.6-fold and 1.7-fold, respectively).

Taken together, measurements on adipocytes confirm a complete GLUT4 translocation inducing properties of purslane and tindora. Comparable to experiments performed on CHO-K1 cells, PP60 treatment induces GLUT4 translocation based on quantitation of the GFP-signal. However, plasma membrane insertion is likely to fail as observed in CHO-K1 cells, since intracellular glucose levels do not increase in PP60 stimulated 3T3-L1 cells.

### Purslane, tindora and ginger extract stimulate GLUT4 translocation mainly through the PI3K pathway

**Fig 4:**
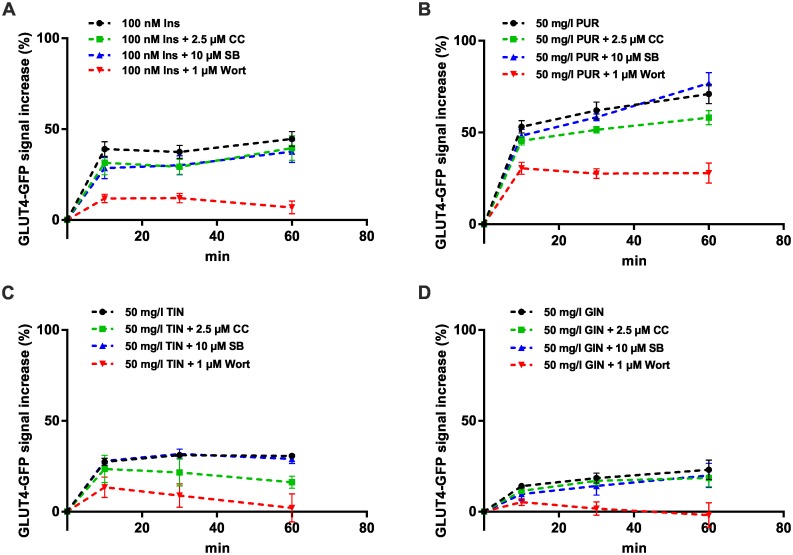
Effects of PI3K, MAPK, and AMPK inhibitors on GLUT4 translocation in PUR, TIN, and GIN stimulated cells measured by TIRF microscopy. CHO-K1 hIR/GLUT4-myc-GFP cells were seeded in 96-well plates (35,000 cells/well), grown over night and then starved for 3 hours in HBSS buffer followed by addition of insulin or the herbal compounds in combination with different inhibitors (CC, SB, wort) for 1 hour. Response curves were generated by measuring the increase of the GFP-signal at 488 nm excitation after application of insulin (A), PUR (B), TIN (C) or GIN (D) at indicated time points. Error bars are based on the standard error of the mean.

To identify the pathway through which the herbal compounds that were found to increase intracellular glucose levels stimulate GLUT4 translocation, CHO-K1 GLUT4-myc-GFP were treated with PI3K, MAPK and AMPK inhibitors, respectively. Then the cells were incubated with purslane, tindora and ginger extract and the increase of the GFP-signal in the evanescent field in the same cells was quantitated (n > 30). As shown in Fig 4A–4D the PI3K inhibitor wortmannin led to the strongest inhibition of the GFP-signal increase for all tested herbal compounds as well as insulin. We found only minor effects in cells pretreated with the MAPK inhibitor SB2013580 and the AMPK inhibitor Compound C, respectively. These results suggest that the PI3K pathway is responsible for the herbal compound-mediated induction of GLUT4 translocation.

### Effects of herbal compounds on actin remodeling

**Fig 5:**
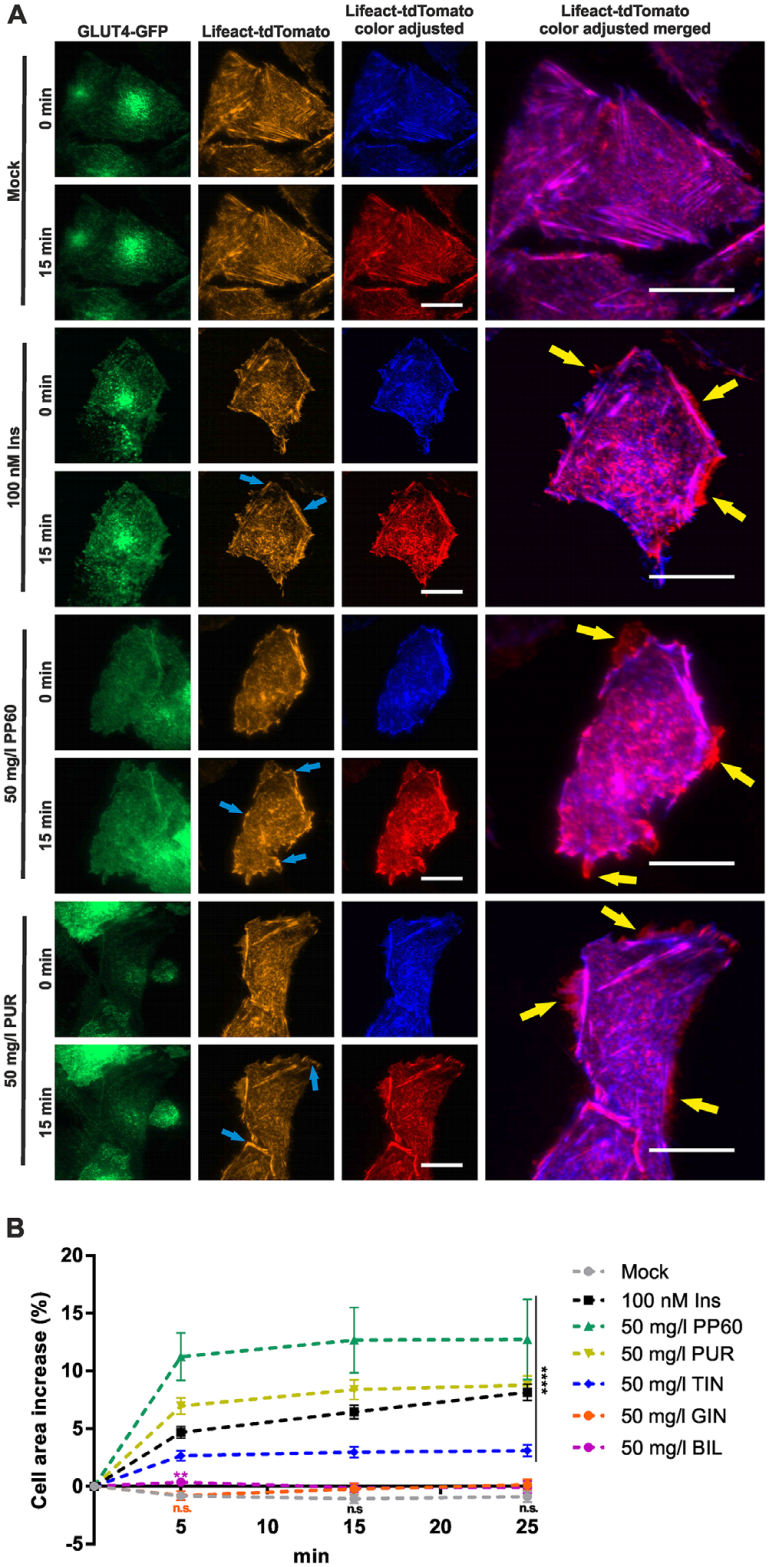
Effects of herbal compounds on actin remodeling. CHO-K1 hIR/GLUT4-myc-GFP cells were transiently transfected with the F-actin marker Lifeact-tdTomato, seeded in 96-well plates (100,000 cells/well), grown over night, and then starved for 3 hours in HBSS buffer. (A) GLUT4-GFP and Lifeact-tdTomato signals recorded at 488 and 561 nm before and after stimulation (15 minutes). For a better visualization of changes in cell size upon compound treatment, additional color adjustment (blue and red images, respectively) of the Lifeact-tdTomato channel is shown. Signal oscillations and sustained intensity increases in the cell periphery are marked by blue arrows, while changes in cell size are indicated by yellow arrows. Scale bar = 20 μm. (B) Quantitation of compound induced change of cell size. Fluorescence images in the Lifeact-tdTomato channel (561 nm excitation) were recorded before and after (5, 15, and 25 minutes, respectively) compound treatment. Size of individual cells was quantitated (n > 30) at the different time points. Error bars are based on the standard error of the mean. **P < 0.01 and ****P < 0.0001, significant increase with respect to mock control.

Insulin has been shown to promote actin polymerization and to increase the rate of actin remodeling [34, 35]. Utilizing a fluorescent F-actin marker (Lifeact-tdTomato) [36] we investigated the effects of selected herbal compounds on actin remodeling. TIRF microscopy has been successfully used to determine cortical actin structures in the immediate vicinity of the plasma membrane [37]. As shown in Fig 5A, when compared to treatment, insulin stimulation (100 nM, 15 min) was found to significantly increase cortical actin structures in the immediate vicinity of the plasma membrane. This sustained increase in intensity level (blue arrows) was accompanied with expansion of the cell (yellow arrow), which could also be detected in the GLUT4-GFP channel. We quantitated the insulin mediated increase in cell size based on the analysis of > 30 cells and found a mean gain of ~6.5% (Fig 5B). The same approach was used to unravel the effects of selected herbal compounds (Fig 5A and S2 Fig). Incubation with PP60 and purslane extract resulted in a mean cell size increase of ~13% and 8.5%, respectively. Tindora extract led to a minor increase, while ginger and bilberry extract did not change the cell size and shape within the investigated time period (15 min). Similar to insulin, PP60, purslane, and tindora extract treated cells were characterized by changes in cortical actin structures near the plasma membrane. In conclusion, PP60, purslane, and tindora extract induce GLUT4 translocation based on a process that involves actin remodeling identical to the effects of insulin.

### Purslane and tindora extract reduce blood glucose levels *in-ovo*

**Fig 6:**
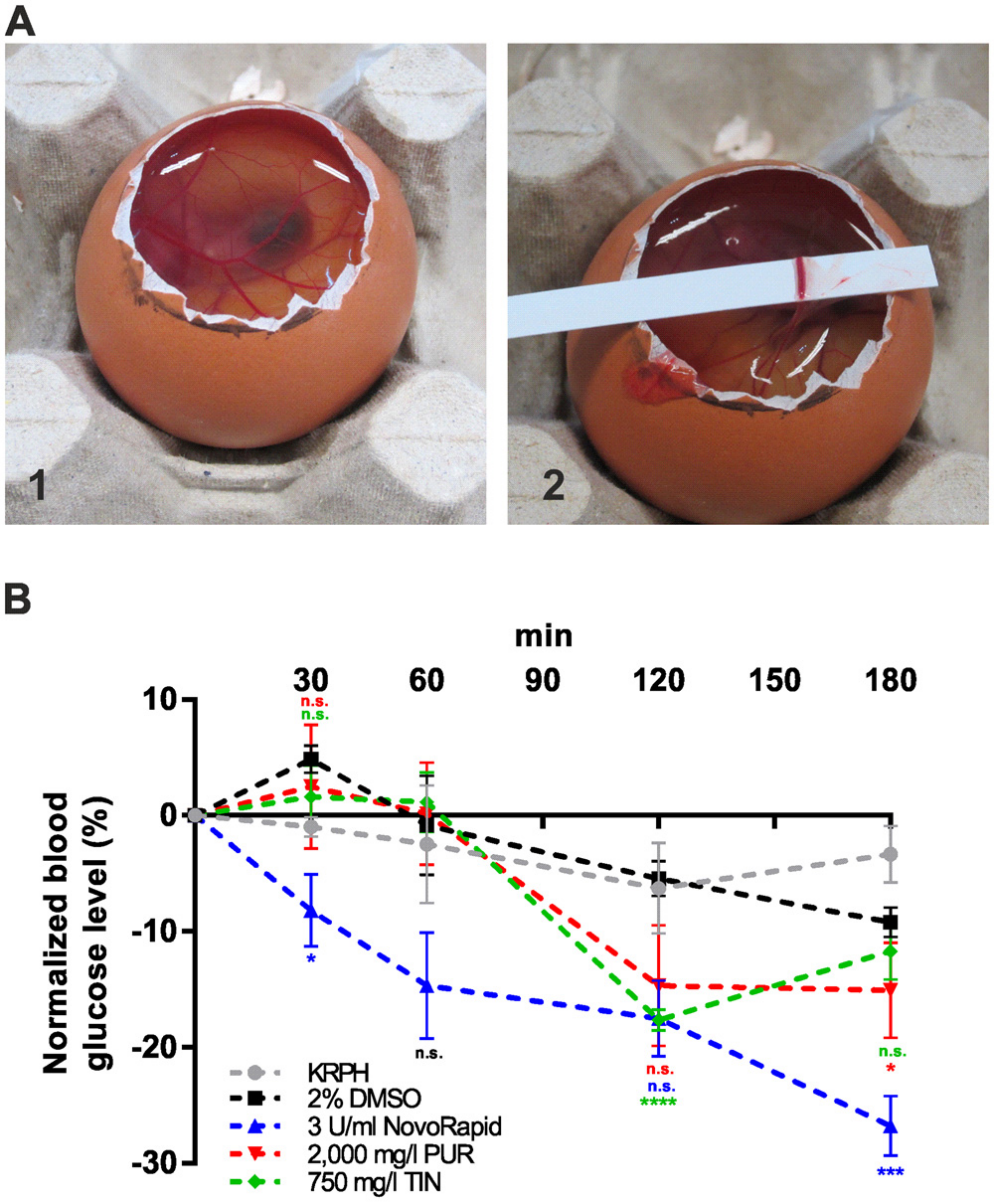
HET-CAM experiments for the determination of blood glucose decreasing properties of selected herbal compounds *in-ovo*. (A) The chorioallantoic membrane of chicken embryos was incubated with the tested substances for 3 hours. After removal of the egg membrane (1), blood was collected from a main vessel (2), and the concentration of glucose in prepared plasma samples was measured by HPLC. (B) Normalized glucose concentration after incubation at indicated time points. Error bars are based on the standard error of the mean. *P < 0.05, ***P < 0.001 and ****P < 0.0001, significant decrease with respect to KRPH (NovoRapid, PUR) or DMSO (TIN) treated cells.

To determine putative effects of the tested herbal compounds on the concentration of blood glucose in a living organism, we used the HET-CAM approach (Fig 6A), which is not regarded to be an animal experiment. The chicken embryo, which is characterized by high glucose levels, has been shown to be an optimal model to study the effects of insulin mimetic drugs on blood glucose levels in a living system [38]. In a first attempt we used the human insulin analog NovoRapid to prove the insulin sensitivity of the test system. As shown in Fig 6B we found a strong decrease of ~27% after 3 hours, while mock treatment (KRPH buffer) resulted in no significant change of the blood glucose level. Based on the previous *in-vitro* experiments, two plant extracts (tindora and purslane) were investigated for their efficacy to decrease blood glucose levels. Incubation with purslane extract resulted in a significant decrease of ~15% and a maximum effect after 3 hours. The first response was detected after 120 minutes. Incubation with tindora extract reduced the blood glucose concentration by ~18% after 120 minutes. Due to the presence of 2% DMSO in the tindora extract, we performed an additional mock control using the same DMSO concentration, and found only a minor decrease of 5–9% after 2–3 hours. Taken together, our HET-CAM experiments confirmed the insulin mimetic properties of tindora and purslane extract *in-ovo*.

## Discussion

The uptake of glucose into muscle cells and adipocytes via GLUT4 has been identified as the rate-limiting step in diabetes [39]. Unfortunately, pharmacological research has not managed to develop an insulin mimetic agent that specifically stimulates glucose uptake. As an alternative, numerous natural compounds have been considered to be antidiabetic substances that could be of benefit in this regard [40]. For this study we investigated several promising antidiabetic plant-derived compound mixtures and focused on their GLUT4 translocation inducing properties. Therefore, a well-established simple cell model—CHO-K1 cells stably overexpressing the human insulin receptor and a GLUT4-myc-GFP fusion protein—was used for primary characterization [41]. In combination with TIRF microscopy it offers the possibility to quantitate GLUT4 translocation at competitive throughput rates in live cells based on the increase of GFP fluorescence intensity in the evanescent field [29]. Six different plant extracts (purslane, tindora, ginger, bilberry, jiaogulan, and milk thistle) as well as a mixture of different polyphenols (PP60) were studied in detail. Incubation with purslane extract or PP60 resulted in a strong increase of the GFP-signal, comparable to or even higher than the one of insulin. Tindora and ginger extract led to a minor increase, while the other tested substances did not stimulate GLUT4 translocation. One of them, namely milk thistle, slightly inhibited GLUT4 translocation upon incubation for 60 minutes, while short time incubation did not result in a significant change. This is consistent with a previous report performed with silybin, the major flavonolignan of milk thistle [42]. In this study the expression levels of GLUT4 remained unchanged upon silybin treatment. In general, milk thistle extract is known to consist of about 65–80% silymarin, which is a complex mixture of polyphenolic compounds including seven closely related flavonolignans and one flavonoid [43]. Here we show that translocation of GLUT4 is not affected by an extract containing high levels of silybin. The positive effects attributed to milk thistle, such as protection against insulin resistance progression [25], are thus likely to be caused by mechanisms independent of GLUT4 translocation. A similar characteristic was found for cells treated with an extract prepared from jiaogulan, a wild growing herb in regions of Asian countries including Vietnam and China. Jiaogulan has been shown to contain high levels of saponins (about 2.5% of the dried plant) termed gypenosides [44]. We did not find remarkably changed GLUT4 translocation levels in our cell model. The described beneficial effects of jiaogulan for the prevention and treatment of diabetes [45] are thus not caused by increased GLUT4 levels in the plasma membrane. Other phenomena such as improved insulin sensitivity [46] and increased insulin secretion [26] are likely to be in the spotlight in this regard. Interestingly, we detected the strongest increase of GLUT4 translocation, at least based on the quantitation of the GFP-signal, in cells treated with PP60, an extract containing mixture of polyphenolic compounds with a minimum of 60% total catechins. The effects of polyphenols on GLUT4 translocation are inconsistent: Some of them induce, while others reduce GLUT4 translocation [47]. Our first results suggested a dramatic increase of GLUT4 translocation in our cell model. However, immunofluorescent staining and atomic force microscopy experiments proved that the final step of GLUT4 translocation, plasma membrane insertion, does not occur under these conditions. One could speculate that the required factors for insertion, such as Rab proteins [48] are not available or active in PP60 treated cells.

Treatment with two further extracts resulted in a significant increase of GLUT4 translocation including plasma membrane insertion, as confirmed by myc-staining and AFM measurements. The first extract was prepared from purslane, which is eaten as a leaf vegetable in many countries and has been reported to improve certain anthropometric measures, serum triglyceride levels, and blood pressure in persons suffering from T2DM [49]. Purslane has been reported to contain high levels of omega-3-fatty acids, vitamins, but also phytochemicals such as betalain pigments [50, 51]. A purslane herb extract termed Portusana™, which was used for this study, is promoted as an intestinal glucose absorption reducing, but insulin sensitivity and cellular glucose uptake increasing product (“EFLA 308”, Frutarom). The second extract was prepared from tindora, commonly known as ivy gourd. In traditional medicine, its fruits have been used to treat various diseases, but knowledge on active ingredients as well as mode of action is very limited. Fatty acids, beta-carotenes, flavonoids, and saponins are among the compounds found in tindora plants [52]. Its blood glucose lowering effect [24] might be caused by an inhibition of glucose-6-phosphatase or by the suppression of adipocyte differentiation [53, 54]. In our primary testing system based on the quantitation of GLUT4 translocation and plasma membrane insertion in CHO-K1 cells, incubation with purslane or tindora extract was associated with the strongest response. Therefore, we repeated quantitation of GLUT4 translocation in physiologically more relevant 3T3-L1 cells. In this adipocyte cell line, only treatment with PP60, purslane or tindora extract resulted in elevated GLUT4 translocation (based on the analysis of the GFP signal), while all other tested substances did not stimulate this process. This finding was supported by glucose uptake measurements in the same cell line. Purslane and tindora treatment were found to increase cytosolic 2-DG levels, while ginger, jiaogulan and bilberry incubation left intracellular glucose levels unaffected. Interestingly, treatment with PP60 and milk thistle extract resulted in a significant reduction of 2-DG concentration, which is consistent with the low level of GLUT4 plasma membrane insertion in cells treated with these compounds.

To identify the pathway through which purslane and tindora stimulate GLUT4 translocation, cells were pretreated with various inhibitors before compound stimulation. Our results indicate that the PI3K pathway is mainly responsible for GLUT4 translocation: Treatment with wortmannin resulted in the strongest inhibition of GLUT4 translocation induction. On the contrary, the efficacy of AMPK and MAPK inhibitors was negligible.

AFM was successfully applied to prove complete plasma membrane insertion upon compound treatment. However, this technique was also used to study the distribution and clustering of GLUT4 proteins by force mapping. It offers the possibility to precisely perform force distance cycles at nanometer resolution. Based on the chosen linker to bind the antibody on the tip, we defined a grid size of 7–8 nm for individual force curves to enable binding with all present GLUT4 molecules in a surface area of 250 nm^2^. We detected mostly single unbinding events, but a small fraction of multiple unbinding events was also found. This is consistent with the reported distribution of GLUT4 proteins as mono- and oligomers in the plasma membrane [55]. Based on our experiments the distribution and clustering of GLUT4 molecules appears similar in insulin, purslane, and tindora treated cells, suggesting a related mechanism of plasma membrane insertion and lateral distribution.

We also studied the effects of tested plant extracts on actin remodeling. Using a fluorescent actin marker we could clearly show that purslane and tindora treated cells are characterized by a significant increase of cortical actin structures in the immediate vicinity of the plasma membrane. This effect was highly similar to the one of insulin stimulated cells, while mock treatment left the organization of actin structures unchanged. Interestingly, incubation with PP60, which was found to stimulate GLUT4 translocation without subsequent plasma membrane insertion, also led to an increase of cortical actin and an enhanced cell size. This result suggests that treatment with PP60 induces GLUT4 translocation by a mechanism dependent on actin polymerization and remodeling, as observed in insulin, purslane or tindora treated cells. However, the insertion of GLUT4 as the ultimate translocation step is lacking in PP60 treated cells. Subsequently, glucose uptake is not promoted under these conditions.

Finally, we used the HET-CAM assay to study the potential of purslane or tindora in reducing blood glucose levels in a living organism, not regarded an animal experiment. In comparison to insulin treated embryos, both extracts induced a slower but significant effect within 2–3 hours.

## Conclusions

Taken together, this study characterizes the GLUT4 translocation modulating properties of several plant extracts. Based on our investigations only two substances, purslane and tindora, led to enhanced translocation, plasma membrane insertion, and glucose uptake. This process appears to depend on the PI3K pathway and is linked to changes in actin remodeling. Furthermore, both extracts result in a distribution and clustering of GLUT4 in the plasma membrane similar to the effectiveness of insulin, and reduce blood glucose levels *in-ovo*.

## Supporting Information

S1 Fig Specificity of GFP-signal increase upon PP60 treatment. (A) CHO-K1 GPI-GFP cells were seeded in 96-well plates (35,000 cells/well), grown over night, and starved for 3 hours in HBSS buffer. Fluorescence intensity was measured before and after 10 minutes of stimulation with PP60 in the same cells (n > 200 cells). Scale bar = 20 μm. (B) Increase of GLUT4-GFP and GPI-GFP signal intensity in CHO-K1 cells treated with PP60. Error bars are based on the standard error of the mean. ****P < 0.0001, significant increase with respect to the GLUT4-GFP increase.
(TIF)

S2 Fig Effects of herbal compounds on actin remodeling. CHO-K1 hIR/GLUT4-myc-GFP cells were transiently transfected with the F-actin marker Lifeact-tdTomato, seeded in 96-well plates (100,000 cells/well), grown over night, and then starved for 3 hours in HBSS buffer. GLUT4-GFP and Lifeact-tdTomato signals recorded at 488 and 561 nm before and after stimulation (15 minutes). For a better visualization of changes in cell size upon compound treatment, additional color adjustment (blue and red images, respectively) of the Lifeact-tdTomato channel is shown. Signal oscillations and sustained intensity increases in the cell periphery are marked by blue arrows, while changes in cell size are indicated by yellow arrows. Scale bar = 20 μm.
(TIF)
